# Informed consent for suspension microlaryngoscopy: what should we tell the patient? A consensus statement of the European Laryngological Society

**DOI:** 10.1007/s00405-022-07429-0

**Published:** 2022-07-12

**Authors:** Frederik G. Dikkers, Michel R. M. San Giorgi, Rico N. P. M. Rinkel, Marc Remacle, Antoine Giovanni, Małgorzata Wierzbicka, Riaz Seedat, Guillermo Campos, Guri S. Sandhu

**Affiliations:** 1 Department of Otorhinolaryngology, Head and Neck Surgery, Amsterdam UMC, University of Amsterdam, Meibergdreef 9, 1105 AZ Amsterdam, The Netherlands; 2 Department of Otorhinolaryngology, Head and Neck Surgery, University Hospital Groningen, Hanzeplein 1, Groningen, The Netherlands; 3 Department of Otorhinolaryngology, Head and Neck Surgery, Amsterdam UMC, Free University Medical Center, De Boelelaan 1118, 1081 HV Amsterdam, The Netherlands; 4 Department of Otorhinolaryngology, Head and Neck Surgery, Centre Hospitalier, Luxembourg, Luxembourg; 5 Service ORL CHU Conception, Aix Marseille Université, Marseille, France; 6 Department of Otolaryngology and Laryngological Oncology, University of Medical Sciences, 49 Stanisława Przybyszewskiego, 60-357 Poznań, Poland; 7 Department of Otorhinolaryngology, University of the Free State, Bloemfontein, South Africa; 8 Department of Surgery, Instituto de Laringologia, Fundacion Santa Fe, University Hospital, Bogotà DC, Colombia; 9 National Centre for Airway Reconstruction, Charing Cross Hospital, Imperial College Healthcare NHS Trust, London, UK

**Keywords:** Phonosurgery, Elective suspension microlaryngoscopy, Shared decision-making, Benign laryngeal pathology, Consent process, Consent discussion, Informed consent, Health care provider, Quality modern health service

## Abstract

**Introduction:**

Informed consent for any surgical intervention is necessary, as only well-informed patients can actively participate in the decision-making process about their care, and better understand the likely or potential outcomes of their treatment. No consensus exists on informed consent for suspension microlaryngoscopy (SML).

**Materials and methods:**

Informed consent procedures in nine countries on five continents were studied.

**Results:**

Several risks can be discerned: risks of SML as procedure, anesthesiologic risks of SML, specific risks of phonosurgery, risks of inadequate glottic exposure or unexpected findings, risks of not treating. SML has recognized potential complications, that can be divided in temporary (minor) complications, and lasting (major) complications.

**Conclusion:**

SML is a safe procedure with low morbidity, and virtually no mortality. Eleven recommendations are provided.

## Introduction

Patient consent is required for any medical treatment. For this consent to be legally valid, the patient must be well informed. Therefore, before asking permission for treatment, a doctor should inform the patient about the proposed treatment in a real dialogue. This dialogue should take into account the patient’s personal circumstances, beliefs and priorities. The process by which a healthcare provider educates a patient about the benefits, risks, and alternatives of a particular procedure or intervention is called informed consent.

Only well-informed patients can actively participate in decision-making about their care: only then can they properly understand the likely or potential results of their treatment. Patient-centered care is, therefore, widely recognized as the core feature of high-quality modern healthcare. When informed consent and the subsequent process of shared decision-making are performed well, damage is prevented, trust is built and surprises and fears are reduced in the event of complications or adverse events. It is evident that no surgical procedure is without risk. Therefore, the benefits, risks and alternatives should be discussed thoroughly with the patient. In the case of minors or adults who for whatever reason are not legally empowered to decide on such procedures themselves, the legal representative should be the partner in this dialogue.

Electively performed suspension microlaryngoscopy (SML) is a common intervention for laryngologists and can be diagnostic or therapeutic. The goal of such an intervention is usually to improve the patient’s voice (‘phonosurgery’), improve the passage of air or diagnose or treat benign or malignant lesions without the need for neck incisions. The aim of this article is to address the medicolegal aspects of elective suspension microlaryngoscopy and to provide recommendations for points of dialogue for the informed consent process, relating to both benign and malignant laryngeal pathology, in the adult patient. To achieve a better understanding, we have reviewed the literature and guideline documents in various countries. The final aim is to present recommendations concerning informed consent.

## Informed consent for microlaryngoscopy throughout the world

In the USA, the concept of informed consent started in 1905 in a judicial decision concerning a case with an otologic intervention. This laid the foundation for the principle of patient autonomy [1]. This first judicial decision was made with the case of Mohr v Williams [1]. The plaintiff, Ms. Mohr, consented to surgery on her right ear. Once she had been anesthetized, her surgeon, Dr. Williams, determined that her left ear was more seriously ill. Dr. Williams then decided to operate on the left ear only. Ms. Mohr’s hearing further deteriorated as a result of the operation. She sued the surgeon for battery and assault for altering the laterality of the operation without permission. The Minnesota Supreme Court agreed with the plaintiff that the surgeon should have obtained explicit consent before performing surgery on the other ear [2].

The required standard for informed consent is determined per state within the USA. However, the following are the required elements for documentation of the informed consent discussion: the nature of the procedure, the risks and benefits of the procedure, reasonable alternatives, risks and benefits of alternatives, and assessment of the patient’s understanding of these elements [3]. The process of informed consent is nowadays shifting to focus more on communication and less on signatures, so the emphasis of a patient signature as an indication of understanding ahead of an elective procedure is currently being called into question [3].

In Australia, a 3-page document has to be filled in before performing SML [4]. The final step in documenting a patient’s decision about consent for microlaryngoscopy is obtained by completing the document. Its domains consist (in this sequence): interpreter and cultural needs, condition and treatment, risks of a microlaryngoscopy, significant risks and procedure options, risks of not having this procedure, anesthetic, patient consent, doctor statement and, if necessary, interpreter’s statement. The doctor has to undersign the statement ‘I have explained to the patient all the above points under the Patient Consent section and I am of the opinion that the patient/substitute decision maker has understood the information.’ There is no explicit definition of significant risks.

In the Netherlands, the care provider must inform the patient in a clear and comprehensible manner about the intended treatment and be guided by what the patient should reasonably know about the expected consequences and risks of the treatment, the alternatives and the outlook [5]. In doing so, the care provider must ensure that the patient has understood what has been discussed with him. The patient’s consent is required for procedures in execution of a treatment agreement. A signature of the patient is not obligatory. Although it is generally recommended to provide the patient with the best possible insight into what to expect, especially if the potential complications are serious, there is no legal obligation to do so if the probability of complications is less than 1% [6]. The surgeon is obliged to document the statements he has made concerning risks, alternatives, complications and expectations in the patient’s file. However, when it comes to non-invasive interventions, the presumed consent of the patient is enough.

In Colombia, informed consent is mandatory for any in-office or operating room procedure, no matter what the magnitude of it might be. It is regulated and overseen by health authorities. Every possible adverse event must be clearly specified in the document, that includes known risks related to SML, any specific potential risks or complications secondary to the use of technologies such as laser, and personalized risks (depending on the clinical conditions of a specific patient), and a final statement about the fact that all questions the patient might have, were satisfactorily answered. An independent informed consent must be signed for the anesthetic procedure.

In France, there is an information document published by the *Centre National Professionnel ORL* which describes minor and major complications. It is recommended that this document be given to patients who require SML. A statement in the file is not mandatory; it is highly recommended to have one.

In Luxembourg, the procedure and the name of the surgeon must be clearly mentioned. The consent is provided in three languages, reflecting the local population. The patient must confirm that he (she) was informed about the objectives, the possible emergency, the possible complications, the alternatives for the treatment, and the risks of not performing the treatment. Acceptance or refusal of blood transfusion must be mentioned in any intervention. Translation in a fourth language must be provided if necessary.

In South Africa, information should be provided to the patient during the informed consent process in a language that the patient understands and should include details of the diagnosis, prognosis, purpose of the procedure, and discussion of any serious or frequently occurring risks. Consent should be obtained by the health care practitioner undertaking the procedure, but where this is not practicable, the task can be delegated to a health care practitioner who is suitably educated, trained and qualified, has sufficient knowledge of the proposed procedure and understands the risks involved. Written consent must be obtained for all cases where the treatment or procedure is complex or involves significant risks and/or side effects, such a SML [7].

In the United Kingdom, based on guidance from the General Medical Council, for consent to be valid, it must be voluntary and informed, and the person consenting must have the capacity to make the decision. This means the patient must understand the information given to them and can use it to make an informed decision. Therefore, the information given to the patient must be clear, accurate and up-to-date, based on the best available evidence. Discussion should include the potential benefits and any risks of harm that the patient would consider significant. In the UK ‘implied’ consent is used for most ‘in-office’ laryngeal examinations but SML requires ‘written’ consent, obtained by a clinician who understands and can perform the intervention.

It is clear that deviations of these protocols exist worldwide. In the nine countries where the procedure of informed consent was examined, laryngologists in just one country have to discuss the informed consent of anesthetics themselves. Details highlighting mandatory demands can be found in Table 1.

**Table 1:** Shortened and summarized details on laryngologist’s mandatory informed consent for suspension microlaryngoscopy in nine different countries. Items that are not mandatory but recommended have not been included

	USA	Australia	The Netherlands	Colombia	France	Luxembourg	South Africa	United Kingdom	Poland
Explanation in patient’s language		+		+		+	+		+
Nature of procedure	+	+	+	+	+	+	+	+	+
Risks of procedure	+	+	+ ^1^	+ ^2^	+	+	+	+	+
Benefits of procedure	+			+			+	+	+
Outlook of intervention			+	+			+		
Reasonable alternatives	+		+	+		+	+		+
Risks and benefits of alternatives	+	+		+			+		+
Anesthetic risks		+							
Any specific risks of technologies such as laser				+			+	+	+
Personalized risks		+		+			+	+	+
Acceptance or refusal of blood transfusion						+			+
Assessment of the patient’s understanding of these elements	+		+	+			+	+	
Patient consent		+	+	+		+	+	+	+
Doctor statement in file		+	+	+			+	+	+
Interpreter and cultural needs		+					+		
Interpreter’s statement in file		^3^							
Written consent/patient signature	^4^	+	–	+	–		+	+	+

^1^Although it is generally recommended to provide the patient with the best possible insight into what to expect, especially if the potential complications are serious, there is no legal obligation to do so if the probability of complications is less than 1%.

^2^Every possible adverse event must be clearly specified in the document, that includes known risks related to SML, any specific potential risks or complications secondary to the use of technologies such as laser, and personalized risks (depending on the clinical conditions of a specific patient), and a final statement about the fact that all questions the patient might have, were satisfactorily answered

^3^If applicable

^4^Being called into question (status 2022)

## Risks of suspension microlaryngoscopy as procedure

SML is a widely used, minimally invasive surgical technique that was initially developed for treatment of problems of the vocal folds. Experience has grown through the years and technological advances have made it possible to expand frontiers to treat pathologies in the pharynx and upper airway, including those that are potentially obstructive. It has inherent risks, even if performed electively. These risks include dental injuries, numbness of the tongue, tongue pain, worsening of the voice, dysgeusia, paresthesia, paresis, and dysphagia. Trauma of the lips, submandibular gland swelling, anterior dislocation of the temporomandibular joint, and rare cases of subcutaneous emphysema have also been reported. Unilateral or bilateral paresthesia, and/or dysgeusia have been attributed to lingual nerve injuries [8]. Rarely, systemic complications arise, such as potentially fatal asystole [9].

In a prospective study of 150 consecutive patients, the most common complications of SML were temporary sore throat, occurring in 66% of patients, and temporary tongue and dental discomfort [10]. In a series of 120 patients, however, only 6 patients (9.5%) experienced postoperative tongue symptoms [11]. Suspension force and duration of suspension were not significantly predictive of postoperative tongue symptoms. While all symptomatic patients were current or former cigarette smokers, smoking status was not found to be a statistically significant factor [11].

The relationship between forces used during SML and tongue-related symptoms has been prospectively analyzed. The maximum force has been stated to be predictive of the development of postoperative complications [12]. Female patients were considered to be at greater risk for developing postoperative tongue symptoms [13]. Others found a relationship with BMI, neck circumference and full mouth opening, whereas some found no gender-based differences of any kind [14].

Prolonged SML (> 220 min) leading to tongue edema requiring close airway monitoring, has been described in two cases [15]. Others also found that the duration of suspension laryngoscopy, not the pressure, had the most significant correlation with postoperative throat pain [14]. Patients whose operations lasted longer than 1 h were almost 4 times more likely to develop tongue-related symptoms than those with an operative time less than 30 min [16]. Remarkably, the opposite has been reported in one prospective study, where tongue compression time had no influence on measured post-SML taste scores [17].

One retrospective series of 213 interventions in 174 patients, all limited to a maximum of 30 min suspension, reported 4 patients with tongue-related complications, 2 oral mucosal alterations, 1 dental injury, and 1 minor facial burn [18]. In a retrospective series of 550 patients at a facility specializing in voice disorders [19], complaints and complications were identified in 66.0% of the subjects; sore throat was reported by 40.0%, which was the highest rate of complaints among all subjects. Tongue-related complications (numbness of the tongue, taste disorder, tongue pain, and hypoglossal nerve palsy) were observed in 16.9% of all cases. A median duration of 4 days was required to recover from tongue numbness or taste disorders. All cases recovered eventually. Almost all complaints and complications were temporary. There were only three cases (0.55%) of tooth damage, which were irreversible. No other serious complications were observed [19].

## Anesthesiologic risks of suspension microlaryngoscopy

Anesthesiologic risks depend on the ventilation method. In a retrospective analysis of 1093 SML procedures [20], ventilation was supplied by mechanical controlled ventilation via small endotracheal tubes (*n* = 200), intermittent apneic ventilation (*n *= 159), transtracheal jet ventilation (*n* = 265), or transglottal jet ventilation (*n* = 469). Twenty-nine minor and 4 major complications occurred. Seventy-five percent of the patients with major events had an American Society of Anesthesiologists physical status classification of III. Five laryngospasms were observed with apneic intermittent ventilation. All other 24 complications (including 7 barotrauma) occurred during jet ventilation [20]. The use of the modern supraglottic high-frequency jet ventilation yielded no severe complications, such as barotrauma, subcutaneous emphysema, or endotracheal fire or death, in both adult or pediatric patients [21, 22].

In most countries, anesthesiologic risks as such have to be discussed with the patient by the anesthesiologist, while in other countries these risks should be discussed by the laryngologist.

## Specific risks of phonosurgery

Phonosurgery is surgery, often performed under SML, with the aim of improving the patient’s voice where there is pathology (nodules, polyps, cysts) or morphological changes (atrophy, scar, paralysis) in one or both vocal cords. There is always the risk of not obtaining the desired voice outcome and this should be discussed with the patient. A discussion should also be had with the patient relating to the individual’s healing biology, which is variable and unpredictable. Vocal irregularities and mucosal rigidity may appear post-surgery, even after a ‘perfect’ surgical intervention. As a result, the possibility that the postoperative voice may, temporarily or permanently, be worse should be part of the dialogue. There should also be a realistic dialogue about the likely period for voice rehabilitation, especially in the ‘professional voice’.

## Risks of inadequate glottic exposure or unexpected findings

The surgeon will have a good idea of expected clinical findings prior to SML. However, the consent should mention the possibility that there may be something unexpected revealed during the examination under general anesthesia. There must be a well-defined agreement between the surgeon and the patient as to the extent to which the surgeon will go to treat unexpected clinical findings. As this may have an impact on long-term voice outcomes or extending the period of recovery, it may be agreed to simply assess the unexpected pathology, wake the patient up and agree a new plan of management with appropriate consent.

While it is certainly an unlikely event, every patient should be aware of the possibility of technical issues during the operation that could impede adequate exposure of the area of interest, or pose a significant possibility for complications, so surgical treatment could be incomplete or not possible at all [23]. There are well-established data concerning pre-treatment predictors of laryngeal exposure, which has been classically regarded as a key limiting factor in determining suspension laryngoscopy feasibility and efficiency [23, 24].

The question remains whether the consent to the procedure should differ significantly in the case of patients with extremely unfavorable anatomy: some surgeons even routinely counsel their patients about the possibility of tracheostomy should acute airway obstruction occur.

## Risks of not treating

Doctors must try to find out what matters to patients so they can share relevant information about the benefits and harms of proposed treatments and reasonable alternatives, including the option to take no action.

With regard to surgery for dysphonia, there are subjective (Voice Handicap Index, GRBAS) and objective (acoustic analysis) assessment tools that can be utilized to make comparisons of the pre-treatment and post-treatment (surgery, medical treatment or speech therapy) voice. It is also important to take into account the patient’s profession, voice commitments and whether the patient has realistic expectations of the outcome of surgery. Equally, the patient may have co-morbidities that make general anesthesia too ‘risky’. These factors and the fact that not all dysphonia requires treatment, may lead to a conservative management plan.

There will be circumstances where, following examination ‘in-office’, the diagnosis is uncertain and a neoplasm cannot be ruled out or staged. In cases with a compromised airway, there is always the potential for this to become worse and increase the possibilities of complications and even death. Providing the patient understands the implications of declining treatment, and has the capacity to make such a decision, then the clinician should respect this. There may then be the option of close monitoring or medical treatments to consider and the patient may have a change of mind in due course.

Doctors must start from the presumption that all adult patients have capacity to make decisions about their treatment and care. A patient can only be judged to lack capacity to make a specific decision at a specific time, and only after assessment in line with legal requirements. The choice of treatment or care for patients who lack capacity must be of overall benefit to them, and decisions should be made in consultation with those who are close to them or advocating for them.

## Discussion

**Fig. 1:**
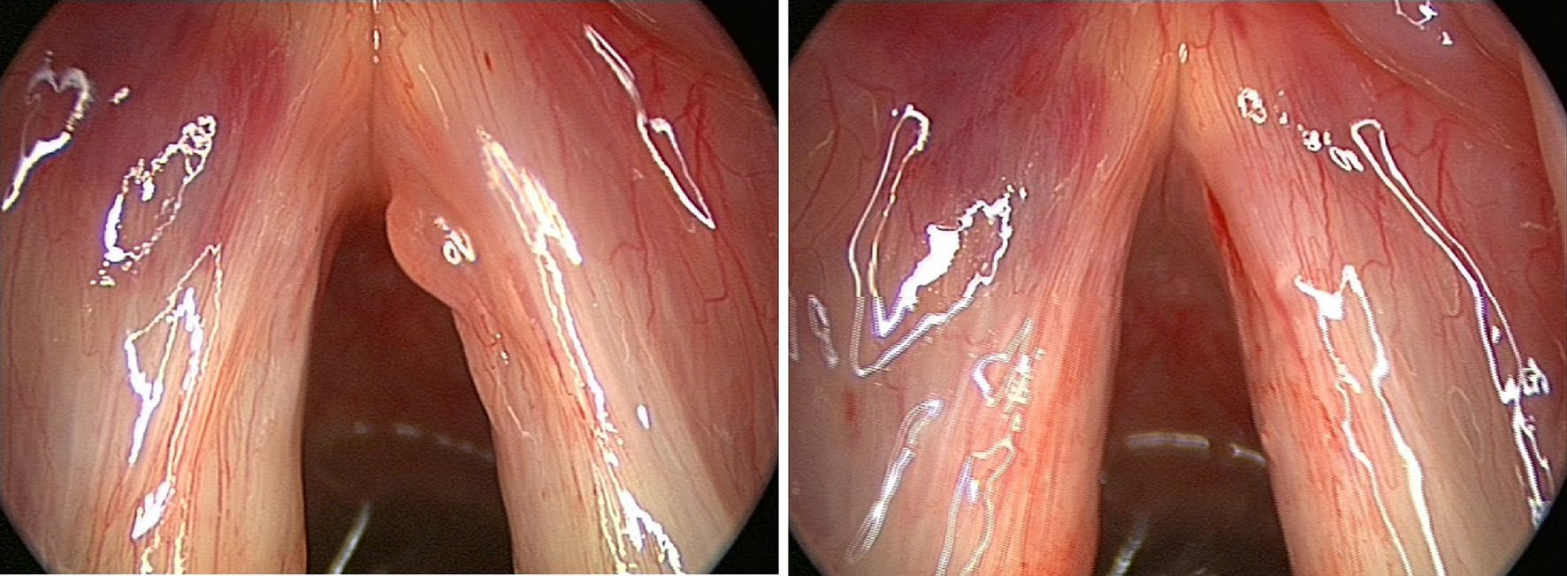
Post-intubation hematoma anterolaterally on the left vocal fold, developed during suspension microlaryngoscopy for polyp on right vocal fold, before (left) and slightly larger and darker after (right) surgery. The hematoma healed without sequelae

**Fig. 2:**
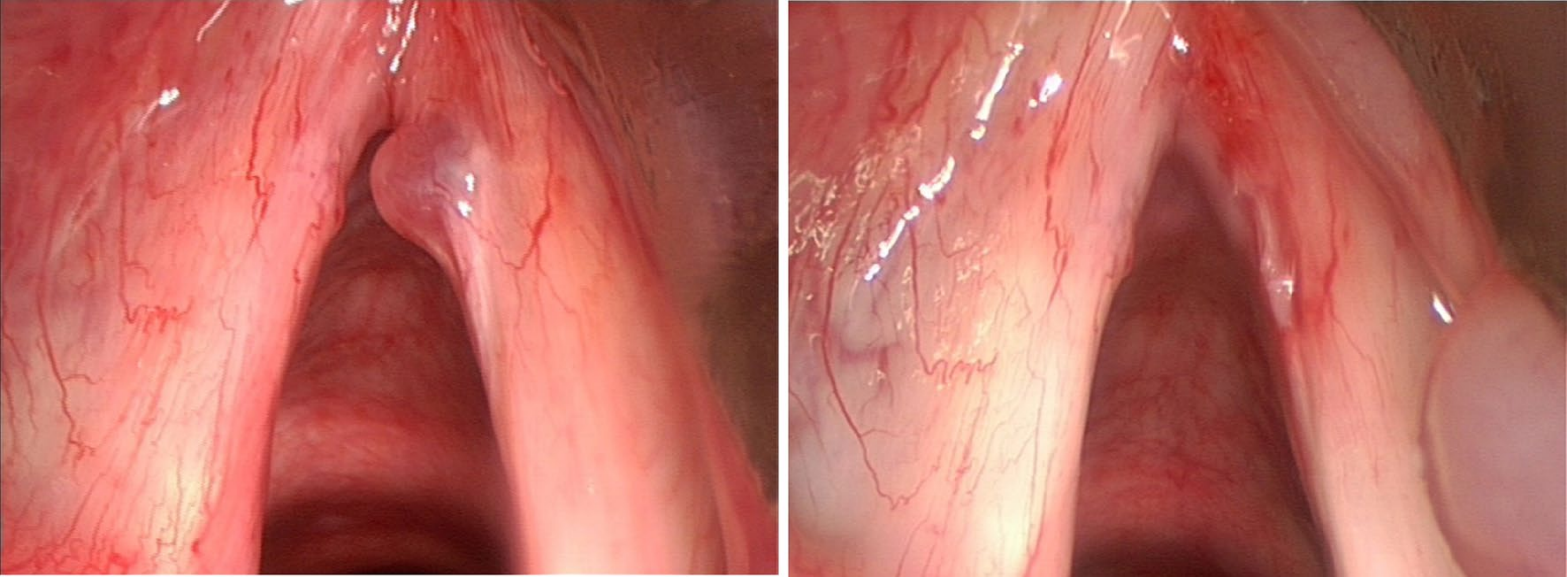
Development of hematoma of the right aryepiglottic fold (bottom right) during suspension microlaryngoscopy for polyp on right vocal fold, before (left) and after (right) surgery. The hematoma developed unnoticed during the intervention, and healed without sequelae

**Fig. 3:**
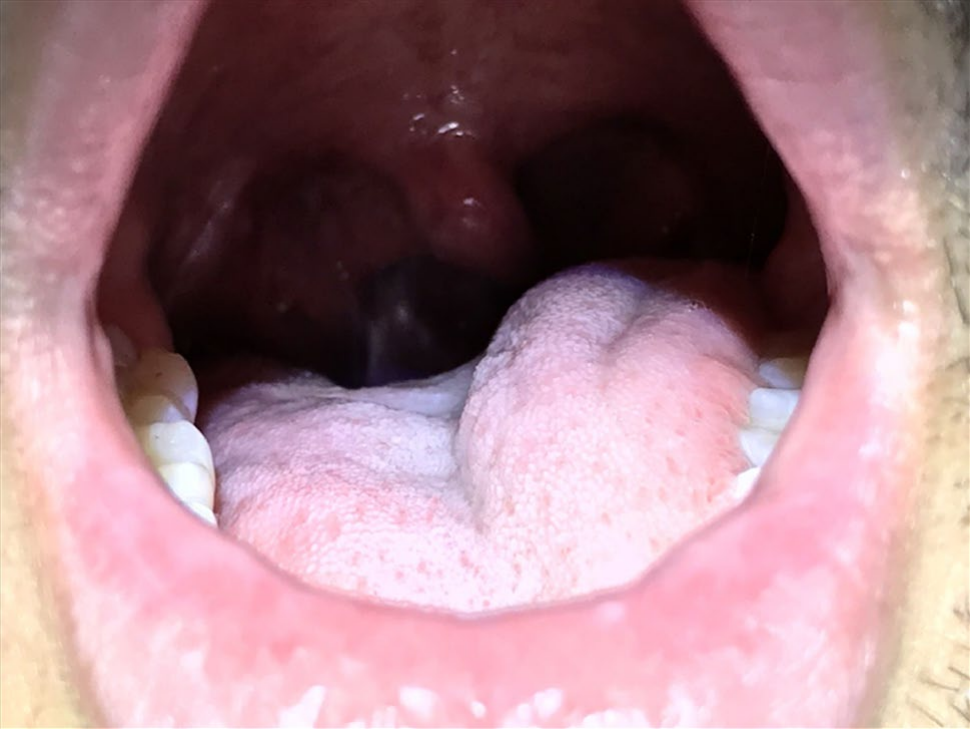
Healthy patient with a transient left hypoglossal nerve palsy immediately after a suspension microlaryngoscopy for treatment of recurrent respiratory papillomatosis

**Fig. 4:**
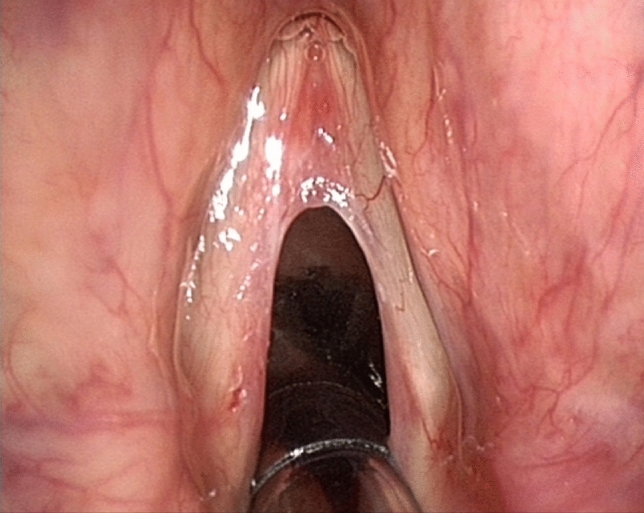
Anterior commissure web formed after suspension microlaryngoscopic surgery, immediately before treatment

SML has recognized complications, that can be divided in temporary (minor) complications, and lasting (major) complications. Complications such as pain, mucosal bruising, post-intubation hematoma (Fig. 1), epiglottic hematoma (Fig. 2) and many tongue symptoms (Fig. 3) resolve spontaneously after hours or days, and are therefore minor complications. The authors are convinced of the fact that these minor complications should be shared with the patient and documented in the patient’s file (according to legal regulations in some countries), even if this does not lead to change in postoperative care. Dental fractures and loss, permanent tongue symptoms and anterior commissure web formation (Fig. 4) are lasting or major complications, or complications requiring corrective interventions.

From a medical-legal standpoint, all possible complications, whether minor and transient, or serious and long lasting, should be part of the consent process, including co-morbidities that may increase the risk of cardiopulmonary events. There should also be a discussion around expected and unexpected outcomes and the likely duration of recovery and voice rehabilitation, as well as statement regarding the need for the patient to comply with all indications and treatment protocols.

‘Risk’ can be regarded as ‘possibility times consequence’. A chance of developing a mucosal bruising in the oropharynx after SML might be 10%. However, nearly all such injuries resolve, hence the risk is small. The chance of a cardiac arrest during SML might be 0.1%; however, the consequences could be severe.

The consent process should include the potential benefits and any risks of harm that the patient would consider significant.

## Conclusions and recommendations

In the context of an experienced laryngology service with a high caseload of predominantly benign pathology, SML is a very safe procedure with low morbidity, and virtually no mortality. As with any surgical intervention, there can be poor outcomes or complications and the patient should be made aware of frequent or significant risks as part of the consent process.

A good outcome after the intervention cannot be guaranteed, the final result of the quality of the voice, the patency of the airway or satisfactory control of a neoplastic growth cannot be predicted. There will always be the possibility of unpredictable situations that are beyond the control of the surgical team.

For a consent process to be valid, it needs to be voluntary and the patient needs to be informed of frequent and significant risks. The information needs to be provided in ‘language’ that the patient understands and the patient must have the capacity to make this decision. Depending on the country, the consent for suspension microlaryngoscopy will be in written form, and the clinician taking the consent must be someone who understands and ideally is trained to perform the procedure. In hospitals with residents this latter scenario is, of course, difficult to reach.

Points to discuss with the patient for suspension microlaryngoscopy:

- What does the surgical procedure consist of, what are the benefits, what are the possible alternatives, what are the risks of the intervention and the recovery process, what is expected from the patient regarding his/ her involvement in postoperative care;
- Required period of voice rest and outline of staged return to normal vocal use if appropriate;
- General anesthesia: discussion of the ventilation mode (intubation, jet ventilation or high flow oxygen) and any associated concerns (depending on the country, as in most countries this belongs to the anesthesiologists’ informed consent);
- Expected period of hospital stay (day case procedure or indication of days);
- Small risk of bruising or cuts to lips, tongue or throat, pain in the temporomandibular joints and numbness, altered sensation or taste related to the tongue, temporary swelling of the submandibular glands (moderate possibility, but usually temporary, thus minor consequence);
- Possibility of permanent voice problems related to surgery;
- Permission to proceed in the event that there is unexpected pathology identified: clarification of the extent to which the surgeon has permission;
- Explanation of the small possibility that it may not be possible to safely achieve adequate surgical exposure and the procedure may be abandoned;
- Serious risk of dental damage (small possibility, major consequence);
- Possibility of hospitalization in the ICU to monitor the airway after procedures for treatment of complex laryngotracheal stenosis without tracheostomy;
- Possibility of ending up with a tracheostomy or prolonged endotracheal ventilation in the case of complex airway surgery (small possibility, major consequence).

Good communication and clear explanation will build trust, and this will reduce surprises and distress if, unfortunately, complications or adverse events do occur. Besides, well-informed patients will have a better understanding of the situation and most probably will be proactive about their recovery process.
